# Down-regulation of *ArabidopsisDND1* orthologs in potato and tomato leads to broad-spectrum resistance to late blight and powdery mildew

**DOI:** 10.1007/s11248-015-9921-5

**Published:** 2015-11-17

**Authors:** Kaile Sun, Anne-Marie A. Wolters, Annelies E. H. M. Loonen, Robin P. Huibers, René van der Vlugt, Aska Goverse, Evert Jacobsen, Richard G. F. Visser, Yuling Bai

**Affiliations:** Wageningen UR Plant Breeding, Wageningen University and Research Centre, Droevendaalsesteeg 1, 6708 PB Wageningen, The Netherlands; Laboratory of Virology, Wageningen University and Research Centre, Droevendaalsesteeg 1, 6708 PB Wageningen, The Netherlands; Laboratory of Nematology, Wageningen University and Research Centre, Droevendaalsesteeg 1, 6708 PB Wageningen, The Netherlands

**Keywords:** Late blight, Powdery mildew, Resistance, RNAi, Susceptibility (*S*) gene, *DND1*

## Abstract

Multiple susceptibility genes (*S*), identified in *Arabidopsis*, have been shown to be functionally conserved in crop plants. Mutations in these *S* genes result in resistance to different pathogens, opening a new way to achieve plant disease resistance. The aim of this study was to investigate the role of *Defense No Death1* (*DND1*) in susceptibility of tomato and potato to late blight (*Phytophthora infestans*). In *Arabidopsis*, the *dnd1* mutant has broad-spectrum resistance against several fungal, bacterial, and viral pathogens. However this mutation is also associated with a dwarfed phenotype. Using an RNAi approach, we silenced *AtDND1* orthologs in potato and tomato. Our results showed that silencing of the *DND1* ortholog in both crops resulted in resistance to the pathogenic oomycete *P. infestans* and to two powdery mildew species, *Oidium neolycopersici* and *Golovinomyces orontii.* The resistance to *P. infestans* in potato was effective to four different isolates although the level of resistance (complete or partial) was dependent on the aggressiveness of the isolate. In tomato, *DND1*-silenced plants showed a severe dwarf phenotype and autonecrosis, whereas *DND1*-silenced potato plants were not dwarfed and showed a less pronounced autonecrosis. Our results indicate that *S* gene function of *DND1* is conserved in tomato and potato. We discuss the possibilities of using RNAi silencing or loss-of-function mutations of *DND1* orthologs, as well as additional *S* gene orthologs from *Arabidopsis,* to breed for resistance to pathogens in crop plants.

**Electronic supplementary material:**

The online version of this article (doi:10.1007/s11248-015-9921-5) contains supplementary material, which is available to authorized users.

## Introduction

Potato and tomato are important food crops that are grown worldwide. Diseases such as powdery mildew and late blight, cause substantial economic losses in these crops. Potato late blight, which is caused by the oomycete *Phytophthora infestans*, has been the most devastating potato disease over 150 years (Fry and Goodwin 1997; Haverkort et al. 2008). Currently, the most effective way of controlling the damage by *P. infestans* is by regular application of fungicide to protect both the infected foliage and tubers. The frequent fungicide applications not only raises environmental concerns, but also gives rise to fungicide-resistant strains of *P. infestans* (Altamiranda et al. 2008; Eschen-Lippold et al. 2010; Bengtsson et al. 2013).

The most common method of resistance breeding to late blight is the introgression of major resistance genes (*R*). After the massive outbreak of late blight in the 1850s in potato, efforts to detect *R* genes have been made in the twentieth century. This resulted in identification and use of a number of *R* genes (NBS-LRR genes e.g. *R1, R2, R3,* and *R10*) from the Mexican wild species *Solanum demissum* (Malcolmson 1969; Wastie 1991). The introgression of *R* genes into cultivated potato species through interspecific crosses and backcrosses can be time consuming and associated with linkage drag (Schouten et al. 2006). Resistance mediated by *R* genes is frequently associated with a hypersensitive reaction (HR), as a result of the interaction between the *R* gene and a specific avirulence gene of the pathogen (Lokossou et al. 2010). Due to new virulent races of *P. infestans,* introduced *R* genes were rapidly overcome by the pathogen after only a few years in the field (Vleeshouwers et al. 2011). An option to achieve a broad spectrum, and more durable resistance, is to stack *R* genes against many isolates (Kim and Hwang 2012; Zhu et al. 2012). In recent years, additional late blight *R* genes have been identified and cloned from other *Solanum* species, including *S. bulbocastanum*, *S. stoloniferum*, *S. venturii*, *S. mochiquense*, and *S. chacoense* (Vossen et al. 2011). The cloning and pyramiding of these *R* genes is facilitated by the availability of genome sequences for potato and tomato as well as information on *P. infestans* effectors (Jo et al. 2011; Vleeshouwers et al. 2011; Gebhardt 2013). Also, rotation of potato cultivars carrying different *R* genes is a good option to extend the effectivity of resistance conferred by *R* genes in the field.

Supplementary to the use of *R* genes, in 2010 we proposed a novel breeding strategy based on “loss-of-function of susceptibility genes” for durable and broad-spectrum resistance (Pavan et al. 2010). Plant genes coding for susceptibility factors are named susceptibility genes (*S* genes), which can be functionally divided into immunity-related and immunity-unrelated groups (Hückelhoven et al. 2013). Immunity-related *S* genes play a role in plant defence responses, while immunity-unrelated genes provide entrance, accommodation and nutrients for pathogens. In a recent review (Van Schie and Takken 2014), *S* genes were divided into three groups based on the time of their action. The first group (*S* gene allowing basic compatibility) includes those that provide early pathogen establishment, such as the *Mildew resistance Locus O* (*MLO*) gene, encoding a membrane-anchored protein (Büschges et al. 1997) that is required for susceptibility to powdery mildews. The second group (*S* genes encoding immune suppressors) contains those that interfere negatively with host defense responses such as the *Cellulose synthase 3* (*CESA3*) gene. The *CESA3* (*cev1*) mutant shows constitutively activated JA and ethylene defence signalling pathways. The third group (*S* genes allowing sustained compatibility) covers those involved in feeding of the pathogen, such as the rice *Xa13* and *Xa25* genes. These two genes encode sugar transporters providing nutrients to the pathogen, *Xanthomonas oryzae* (Chen et al. 2010).

From a genetic point of view, we consider a gene as an *S* gene, when impairment of its function can lead to recessively inherited resistance (Pavan et al. 2010). In *Arabidopsis*, many loss-of-function mutations are known to cause pathogen resistance. Examples are powdery mildew resistance (*pmr*; Vogel and Somerville 2000; Vogel et al. 2002) and downy mildew resistance mutants (*dmr* to *Hyaloperonospora parasitica*; McDowell et al. 2005). However, only few *S* genes have been tested for their conserved function in crops. The best-studied example is the *Mlo* gene. Mutations in certain *Mlo* homologs in plant species (e.g. barley *Mlo*, *Arabidopsis AtMlo2*, pea *PsMlo1* and tomato *SlMlo1*) result in disease resistance by preventing the penetration of the adapted powdery mildew species (e.g. Bai et al. 2008; Pavan et al. 2011; Zheng et al. 2013). The first identified barley *mlo* mutant has been successfully used in barley cultivars in European agriculture for more than 35 years, illustrating the durability of *mlo*-based resistance (Lyngkjaer et al. 2000; Humphry et al. 2006).

Our research is aimed at identifying *S* genes for susceptibility to different pathogens in tomato and potato. We have tested the susceptibility function of several *S* genes identified in *Arabidopsis* and demonstrated that in tomato, silencing *PMR* and *DMR* orthologs gives rise to resistance against the mildew fungus *Oidium neolycopersici* (Huibers et al. 2013). Our results suggest that *S* genes such as *MLO*, *PMR* and *DMR* genes are functionally conserved among plant species and that silencing orthologs in other plant species can give resistance to fungi. In this study, we focused on the *ArabidopsisAtDND1* (*Defense No Death 1*) gene (Yu et al. 1998) encoding a cyclic nucleotide-gated cation channel (CNGC; also known as *AtCNGC2*, Clough et al. 2000). In *Arabidopsis*, *dnd1* mutants were reported to show resistance to *Pseudomonas syringae* and other fungal and viral pathogens (Yu et al. 2000; Jurkowski et al. 2004; Genger et al. 2008). Here, we show that silencing tomato and potato *DND1* orthologs resulted in resistance to both late blight and powdery mildew.

## Materials and methods

### Plant growth and cultivation

Tomato (*S. lycopersicum* cultivar Moneymaker, MM), tetraploid potato *S. tuberosum* cultivar Desiree, diploid potato genotype SH83-92-488 (SH) (Rouppe van der Voort et al. 1997) and potato transformant A13-013 carrying the *Rpi*-*vnt1.1* resistance gene in Desiree background (Foster et al. 2009; Pel et al. 2009) were used. Plants were grown in greenhouses at 21 and 19 °C during the 16 h day and 8 h night periods respectively. Relative humidity was 70 % and light intensity was supplemented with 100 W/m^2^ when light intensity dropped below 150 W/m^2^. Potato transgenic plants were also grown in large 5-l pots in an insect-free screen cage on a concrete floor under outdoor summer conditions. All transgenic plants were grown with a GMO permit according to Dutch GMO regulations.

### Identification of tomato and potato DND1 orthologs

The *Arabidopsis thaliana* DND1 (GenBank accession number BAE99132) amino acid sequence was used as a query in a BLASTP program against the Sol Genomics Network (SGN) Tomato Genome protein sequences (ITAG release 2.40) or Potato PGSC DM v3.4 protein sequences database (http://solgenomics.net/tools/blast/) or against an *Arabidopsis* protein database (http://www.arabidopsis.org/Blast/index.jsp) to search for homologous sequences in tomato, potato and *Arabidopsis*, respectively. The alignment of the protein sequences was done by DNASTAR Lasergene 8 and phylogenetic trees were constructed by using Mega4.0 (Tamura et al. 2007). The evolutionary history was inferred using the neighbour-joining method (Saitou and Nei 1987). The percentage of replicate trees in which the associated taxa clustered together in the bootstrap test (10,000 replicates) is shown next to the branches.

### Generation of RNAi transgenic plants

The binary vector pHellsgate 8 (CSIRO, Australia) was used to generate RNAi constructs (Waterhouse and Helliwell 2003; Helliwell and Waterhouse 2003) with a CaMV35S promoter and containing a kanamycin resistance gene as selectable marker. Primers were designed to amplify fragments of the target genes ranging from 150 to 300 bp (Supplementary Table 1). The forward primer contained CACC at the 5′ end for TOPO cloning. Tomato MM cDNA was used as PCR template. The PCR products were cloned into the pENTR/D-TOPO cloning vector (Invitrogen, USA) and were sequenced. The final RNAi vector was produced by an LR clonase reaction between the entry clone and pHellsgate8 vectors. Transformation of MM seeds was carried out as described by Huibers et al. (2013). For potato transformation the protocol described by Visser (1991) was used.

### Nucleic acid extraction and RNA expression analyses

Nucleic acid extraction was carried out as described by Huibers et al. (2013). Quantitive real-time PCR (qRT-PCR) was performed to determine the expression levels of potato and tomato *DND1* in different tissues without pathogen stress conditions. Tomato RNA samples were isolated from 12-week-old MM plants grown in the greenhouse. Potato samples were isolated from 8-week-old Desiree plants from the field. The qPCR was performed in triplicate with a C1000 light cycler system (Bio-Rad) using SYBR Green mix (Bio-Rad) with gene-specific primers for *DND1* (Supplementary Table 1). qRT-PCR data for *DND1* were compared with RNAseq data from potato and tomato. Potato FPKM values for *Sotub02g034320* were obtained from the Spud DB website (http://solanaceae.plantbiology.msu.edu/index.shtml). Tomato RPKM values for *Solyc02g088560* were obtained from http://ted.bti.cornell.edu/cgi-bin/TFGD/digital/home.cgi using library D004: Transcriptome analysis of various tissues in tomato cultivar Heinz and the wild relative *Solanum pimpinellifolium*.

The expression level of *StPR1* (GenBank AJ250136) in Desiree and transgenic potato plants was analyzed by qRT-PCR with RNA samples isolated from 6-week-old plants grown in the greenhouse. In addition, *StPR1* expression was analyzed in selected transgenic plants compared with wild type Desiree after mock inoculation or inoculation of 6-week-old plants grown in the greenhouse with *P. infestans* isolate Pic99189. Gene-specific primers are shown in Supplementary Table 1. Relative quantification was performed by qRT-PCR using the ΔΔCt method (Livak and Schmittgen 2001). The potato and tomato elongation factor 1-a (EF1a, *Sotub06g010680* and *Solyc06g005060*) transcripts were used as the internal control to calculate the relative transcript levels. The gene-specific primers used for development of RNAi constructs, qPCR, and *O. neolycopersici* quantification are shown in Supplementary Table 1. For the qRT-PCR assays, three technical replicates were performed for each experiment and the expression of each gene was investigated in three biological replicates.

### Disease assays

For detached leaf assays (DLA, Vleeshouwers et al. 1999) with *P. infestans*, several isolates were used. Information on virulence factors, mating type and data concerning the collection of *P. infestans* isolates is presented in Supplementary Table 2. These isolates were cultured on rye sucrose medium (Caten and Jinks 1968) in the dark at 15 °C for 10–14 days. Sporulating mycelium was flooded with cold water and the sporangiospore suspension was incubated at 4 °C for 1–2 h. The inoculum was adjusted to a concentration of 5 × 10^4^ zoospores/ml. Potato leaves were taken from plants grown in the greenhouse. The lesion size on leaflets was measured using a calliper with digital display (DIGI-MET^®^, Helios Preisser, Germany) at 3–6 days after inoculation. For potato tuber assays, the protocol described in Zhu et al. (2012) was applied. Tubers were taken from both greenhouse and screen cage-grown plants. Two assays with greenhouse tubers, and one assay with screen cage tubers were performed. For each transformant, four plants were tested as biological replicates.

For the test with tomato powdery mildew, the Wageningen isolate of *Oidium neolycopersici*, On-Ne (Bai et al. 2005) was used which has been maintained on plants of MM. Spore suspensions were obtained by washing heavily infected MM leaves in water. Tomato plants of about 4 weeks old were sprayed with an inoculum of 2.5 × 10^4^ spores per ml. Fungal quantification by qPCR was performed on leaf samples collected between 8 and 14 days post inoculation (dpi). *Oidium* Internal Transcribed Spacer (ITS)-specific primers (Huibers et al. 2013) are shown in Supplementary Table 1.

For the nematode test, *Globodera rostochiensis* line 19 (the Ro1 pathotype) was used. Potato plants were obtained from sprouting tubers in sterile clay pots covered with sterile sandy soil. Four to five weeks after tuber planting the rooting plants were inoculated with the nematodes. To collect the eggs from the cysts, the cysts were soaked overnight in water. Then the cysts were crushed with a special glass pestle. After that, the suspension was filtered over a 100 µm sieve. The final number of eggs for inoculation was around 10,000 eggs per pot. The cysts were extracted from roots 6 weeks post inoculation and counted by following the protocol described in Van Bezooijen (2006).

For disease test with potato virus Y (PVY), the *PVY*^*NTN*^ strain (isolate PRI-757) was used which was maintained in tobacco plants, *Nicotiana tabacum* cultivar Samsun. Potato transformants were regenerated from in vitro plantlets and grown in the greenhouse in soil under controlled environmental conditions (16/8 light/dark cycle, 24 °C) for 4 weeks before *PVY* inoculation. Three newly developed upper leaves were dusted with carborundum powder and rubbed with cheesecloth dipped in a sap prepared from the leaves of the *PVY*-infected tobacco plants. After 10 min, the leaves were washed liberally with tap water. In mock inoculations, water was used instead of the sap. The presence of *PVY*^*NTN*^ in plants was monitored using a polyclonal rabbit antiserum, raised against purified virus, in a double antibody sandwich (DAS) enzyme-linked immuno-absorbent assay (ELISA) (Van den Heuvel and Peters 1989). All plants, both the inoculated plants and the mock non-inoculated plants were checked for the presence or absence of virus 14 dpi by DAS-ELISA. Each treatment had four plants as biological replicates.

### Pathogen identification

To verify the identity of powdery mildew species affecting potato in the greenhouse, DNA was extracted from powdery mildew infected leaves using DNeasy^®^ Plant Mini Kit (QIAGEN, Germany). Fungal ITS primers, ITS5A (5′-TTGGAAGTAAAAGTCGTAAC-3′, derived from ITS5) and ITS4 (5′-TCCTCCGCTTATTGATAGC-3′) were used (White et al. 1990). The PCR products were sequenced and blasted against the NCBI database (http://www.nibi.nlm.nih.gov/BLAST/).

### Time lapse video

A time lapse video was produced to visualize *P. infestans* development on leaves of wild type potato Desiree and transgenic RNAi::DND1A plants. A 12-cm square Petri dish was filled with autoclaved demineralized water containing 1 % agar to a thickness of 5 mm. Detached potato leaflets were transferred into the Petri dish and kept until the medium was solidified. Leaflets were taken from the same plants as those used in the DLA (see above). They were inoculated with *P. infestans* isolate Pic99189 zoospores using the same inoculum concentration as in the DLA (5 × 10^4^ zoospores/ml). Then, the Petri dish was sealed with Parafilm and placed in a closed box containing a Nikon D90 DSLR camera with a Nikon 60 mm lens. To achieve a 16 h light/8 h dark cycle, the Petri dish was illuminated with Philips LED modules (4× white, 2× blue and 2× deep red modules; light intensity 37.9 μE m^−2^ s^−1^) connected to a microprocessor (Arduino Uno) controlling the light. With the help of Nikon Camera Control Pro 2 software, photos were automatically taken once every 15 min. The video is played back at 10 frames per second, and edited using Adobe Photoshop Lightroom 5 software. Depending on the growth conditions such as light and composition of the medium in this experiment, plants can be observed in the Time lapse system for up to 5 days.

## Results

### Identification of tomato and potato DND1 orthologs

Using BLAST analysis we identified a single tomato gene (*Solyc02g088560*) encoding a protein with a high level of amino acid identity (75 %) with AtDND1 (*At5g15410*), and hereafter referred to as *SlDND1*. In potato, a single gene was also identified (*Sotub02g034320*) encoding a protein having 75 and 99 % identity with *Arabidopsis* AtDND1 or tomato SlDND1, respectively (Supplementary Fig. 1a), hereafter referred to as *StDND1*. In *Arabidopsis* there are 20 CNGC genes (Chin et al. 2013). In a protein-based phylogenetic tree SlDND1 and StDND1 showed higher homology to AtDND1 (CNGC2) than to any other *Arabidopsis* CNGC family member (Supplementary Fig. 1b). Therefore, *SlDND1* and *StDND1* are the most likely candidates for being *AtDND1* orthologs.

### Transcript levels of DND1 in various tissues of tomato and potato

Expression levels of *DND1* were examined in leaf, root, flower, fruit (tomato only), and tuber (potato only) samples by quantitative real-time PCR. As shown in Supplementary Fig. 2, *DND1* mRNA was detected in all tissues, but at different levels. The *DND1* expression level was highest in flowers, more than 15-fold as high as that of the leaves. In contrast, *DND1* transcripts were low in roots, tomato fruit or potato tubers (Supplementary Fig. 2a, b). These measured expression levels of *DND1* correlated well with RNAseq data (RPKM or FPKM values) for tomato cultivar Heinz and potato genotype RH (Supplementary Figure 2c, d respectively).

### StDND1 silenced potato plants are resistant to late blight

Two RNAi constructs (RNAi::DND1A and RNAi::DND1B) were made based on the tomato *SlDND1* sequence (Supplementary Fig. 3a). These two constructs were designed to silence both tomato and potato *DND1* orthologs (Supplementary Fig. 3b), with RNAi::DND1A targeting the 5′ part of the coding sequence and RNAi::DND1B targeting the 3′ end.

Both RNAi constructs were used to transform potato cultivar Desiree, and in total 187 transgenic plants were obtained. For each RNAi construct, eight plants were randomly selected and the expression level of *StDND1* was measured (Supplementary Fig. 3c). Compared with Desiree, the expression level of *StDND1* in the transformants was reduced 10–90 %. Three transformants of each construct, DND1A-5, -8 -17 and DND1B-6, -8, -11, which had a high silencing level of *StDND1* (well-silenced and indicated as +) and a plant morphology and growth similar to wild type Desiree, were selected for further experiments. As negative controls, transformants with a low degree of silencing (indicated as −), DND1A-6 and DND1B-4, were selected and included in further analyses. The selected plants were multiplied by in vitro cuttings and tubers were harvested after growth of these cuttings in the greenhouse.

**Fig. 1:**
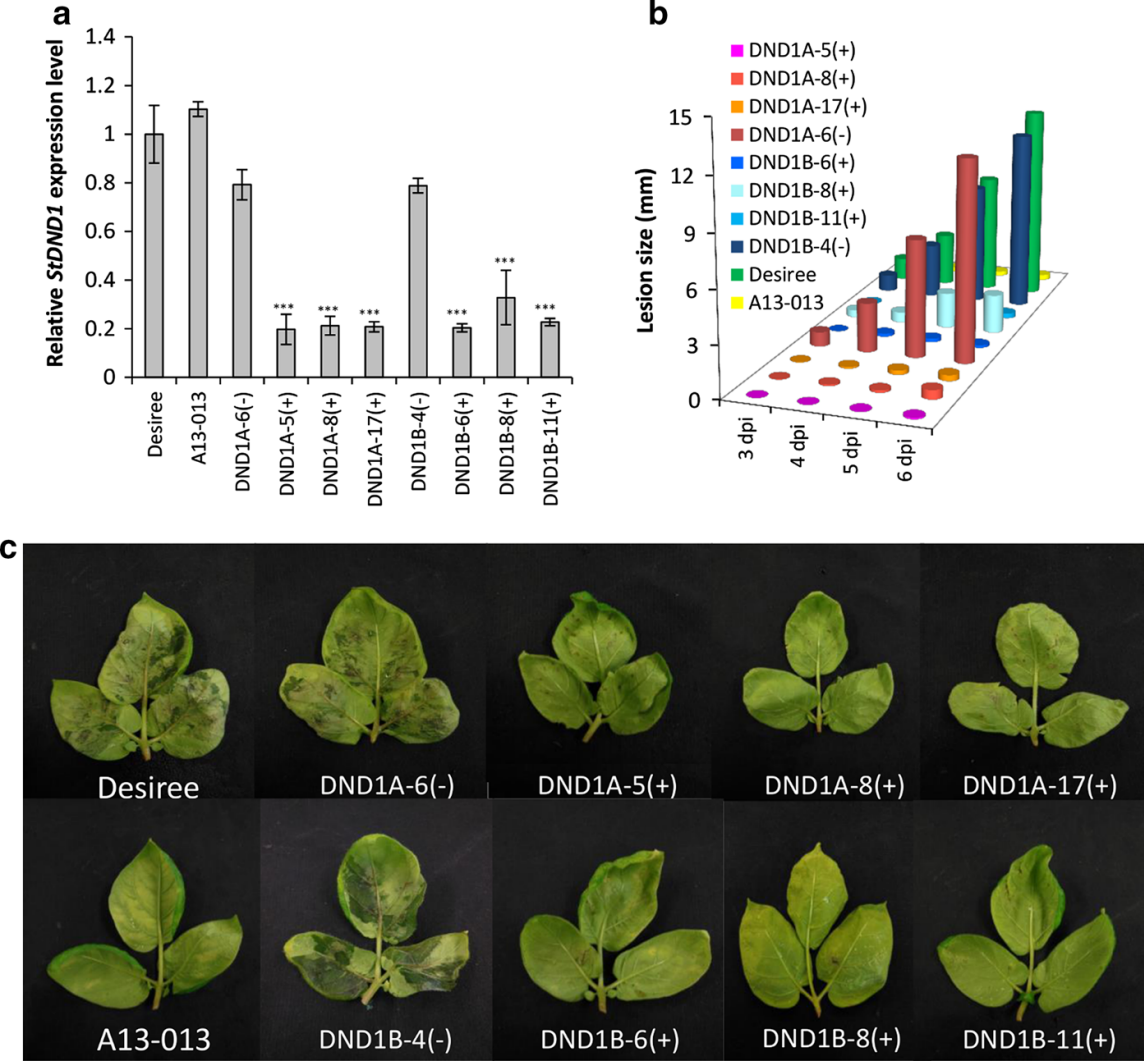
Late blight resistance in potato leaves by silencing *StDND1*. **a** Relative expression level of *StDND1* in leaves of Desiree, A13-013 and eight independent potato RNAi::*DND1* plants (DND1A-5, 6, 8 and 17, DND1B-4, 6, 8 and 11). **b** Development of lesion size on plants infected with *Phytophthora* *infestans* isolate Pic99189. Data were collected at 3, 4, 5 and 6 days post inoculation (dpi). Well-silenced (+) plants showed significantly lower growth rate compared to untransformed Desiree and weak-silenced (−) plants. **c** Pictures of potato leaves taken at 7 dpi. *Note* the same plants were used for all the measurements. For each transformant, four plants were tested (one leaf per plant). Three independent experiments were performed with similar results

**Fig. 2:**
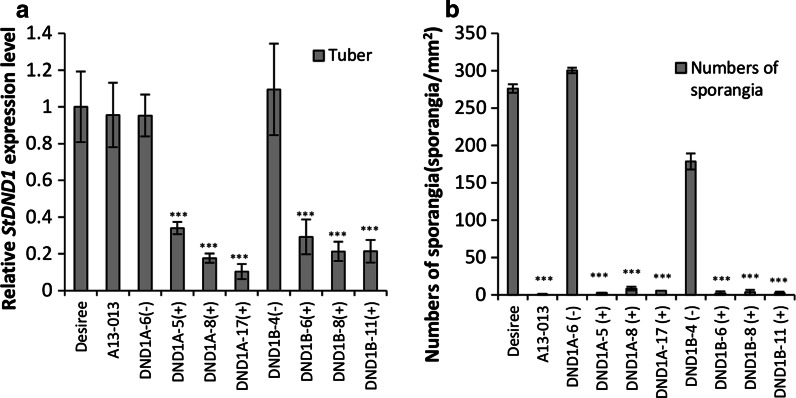
Late blight resistance in potato tubers by silencing *StDND1*. **a** Relative expression level of *StDND1* in tubers of Desiree, A13-013 and eight independent potato RNAi::*DND1* plants (DND1A-5, 6, 8 and 17, DND1B-4, 6, 8 and 11). **b** Numbers of sporangia on tubers infected with *Phytophthora infestans* isolate Pic99189. For each transformant, four plants were tested (one tuber per plant). *Asterisks* indicate degree of significance compared to Desiree plants (***p < 0.001)

To assess the response of the selected plants to *P. infestans* infection, they were inoculated with the isolate Pic99189 in a DLA assay. The expression level of *StDND1* was verified (Fig. 1a). Infection lesion diameter on infected leaves was determined daily from 3 to 6 dpi (Fig. 1b). The resistant control A13-013 carrying the *Rpi*-*vnt1.1* gene showed no lesions during the whole period. In contrast, leaves of the susceptible control Desiree were almost fully covered by late-blight mycelia at 7 dpi. Three DND1A (+) transformants and two DND1B (+) transformants were as resistant as A13-013. Only DND1B-8(+) showed restricted lesion growth and a more variable level of *StDND1* expression compared to the other (+) transformants (Fig. 1a). Plants of the two *DND1* weak-silenced (−) transformants were as susceptible as Desiree (Fig. 1c). Development of symptoms on detached leaves of Desiree and transformants DND1A-6(−), DND1A-5(+) and DND1A-17(+) is also shown in the time lapse movie Supplementary Video 1. The transformants in the DLA assay were also subjected to a tuber slice assay with *P. infestans*. Similar to the DLA results, tubers of the well-silenced transformants showed significantly lower levels of *StDND1* expression (Fig. 2a) and almost no late blight sporulation compared to Desiree and the weak-silenced transformants (Fig. 2b).

For both DLA and tuber assays, three independent experiments were performed with similar results, demonstrating that the RNAi plants in which *StDND1* is well silenced could lead to complete resistance to *P. infestans* isolate Pic99189 in both leaves and tubers. To analyse whether the resistance observed under greenhouse conditions was stable in other environmental conditions, a tuber slice assay was performed using tubers that developed on plants grown in a screen cage under outdoor conditions. Results obtained with these tubers (Supplementary Fig. 4) were similar to those from greenhouse-grown tubers (Fig. 2b).

### StDND1 silenced potato plants show resistance to multiple isolates of late blight

**Fig. 3:**
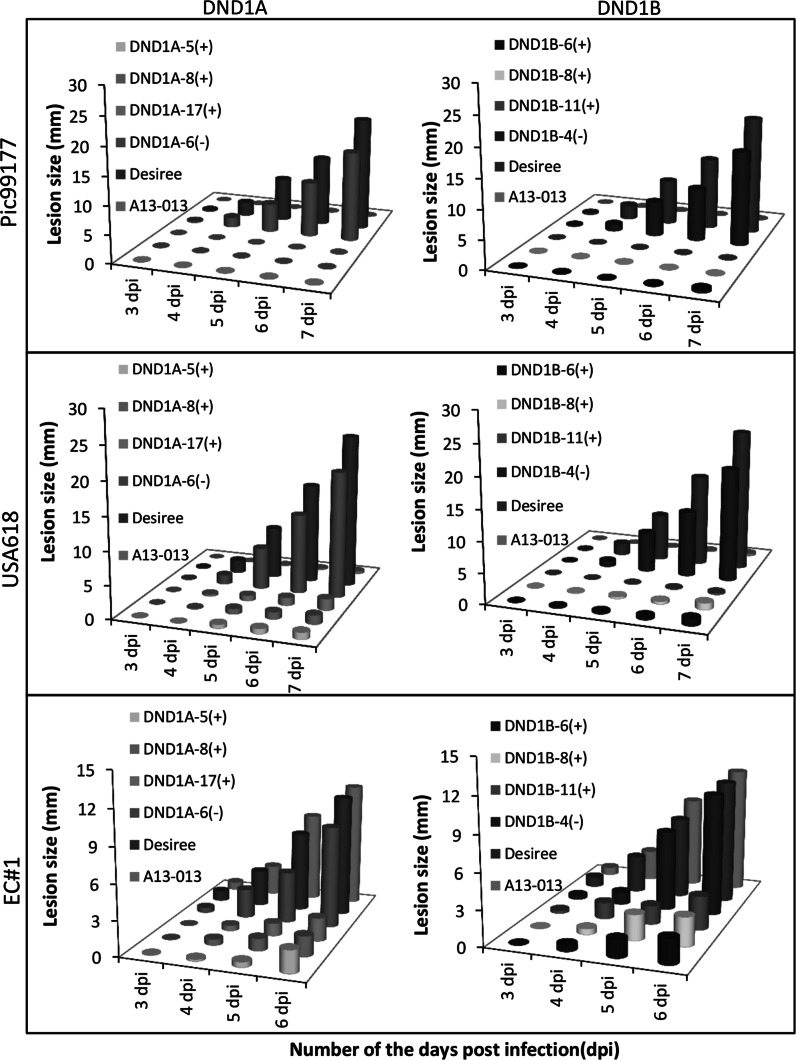
Broad spectrum resistance to late blight in potato by silencing *StDND1*. *Graphs* show the development of lesion size of infected leaves of different genotypes after inoculation with three *Phytophthora infestans* isolates Pic99177, USA618 and EC#1. Cuttings from the same transformants as in Figs. 1 and 2 were used. For each transformant, four plants were used. From each plant, three leaves were detached and each was inoculated with one isolate. The experiment was repeated twice with similar results

In order to investigate whether *StDND1* silenced potato plants show race non-specific resistance to *P. infestans*, the above tested RNAi transformants were challenged in a DLA assay with three additional *P. infestans* isolates; Pic99177, USA618 and EC#1. Desiree was considered as the susceptible control and A13-013 as the resistant control for isolates Pic99177 and USA618. Both Desiree and A13-013 are susceptible to the aggressive isolate EC#1. Development of lesion sizes on the infected leaves was determined from 3 to 7 dpi (Fig. 3). The experiment with the aggressive isolate EC#1 was, however, terminated at 6 dpi because of serious disease symptoms. Similar to the results obtained with the isolate Pic99189, RNAi plants in which *StDND1* is well-silenced, showed resistance to isolates Pic99177 and USA618 (Fig. 3). With the aggressive isolate EC#1 significantly smaller lesion sizes were observed for *StDND1* well-silenced (+) transformants compared to Desiree and A13-013 (Fig. 3). In general, lesion growth on the susceptible plants (Desiree, A13-013 and non-silenced transformants) started from 3 dpi, while DND1A (+) and DND1B (+) transformants showed a delayed and restricted lesion growth starting from 4 dpi. These experiments were performed twice with similar results, showing that the acquired resistance by silencing of *DND1* in potato is not specific to the tested isolates, though the resistance level may partially depend on the aggressiveness of the *P. infestans* isolates.

### StDND1 silenced potato plants are resistant to powdery mildew

When potato plants are grown in the greenhouse they can be naturally infected by powdery mildew (Supplementary Fig. 5a). To verify the identity of the powdery mildew species infecting potato plants, DNA was isolated from infected potato leaves and used as a template for PCR amplification with fungal ITS (Internal Transcribed Spacer) primers (Supplementary Fig. 5b). The sequence amplified in this ITS region was identical to that of *Golovinomyces orontii* MUMH 2003 (GenBank AB427188, Uchida et al. 2009). Sporulation (characterised with a Disease Index (DI) score of about 2.5, Supplementary Fig. 5c) was observed on the upper side of older leaves of Desiree as well as on old leaves of the two weak-silenced transformants, DND1A-6(−) and DND1B-4(−). In contrast, no fungal sporulation (DI score of 0, Supplementary Fig. 5c) was observed on leaves of well-silenced (+) transformants. Natural infection was uniform and plants were randomized. Fungal biomass was quantified (Supplementary Fig. 5d). All *StDND1* silenced (+) plants showed significantly lower fungal biomass compared to the controls (Desiree and A13-013) and *StDND1*(−) plants.

### Resistance by silencing of DND1 is specific to certain pathogens

To test whether *DND1*-silenced plants show resistance to a broad range of pathogens, *StDND1* silenced potato plants were tested with nematode *Globodera rostochiensis* and potato virus Y (*PVY*). Compared with the susceptible control Desiree plants, all *StDND1* silenced potato plants were susceptible to *PVY* (Supplementary Fig. 6). In the nematode test, diploid potato genotype SH carrying the *H1* resistance gene was the resistant control. SH showed almost no cysts growing on the roots, while Desiree showed a high number of cysts (on average 250 per gram dry root weight (Supplementary Fig. 7). A lower number of cysts was observed on the well-silenced *DND1* transformants. This reduction was however in general not significantly different from cyst number in Desiree.

### Silencing of tomato SlDND1 results in powdery mildew and late blight resistance

**Fig. 4:**
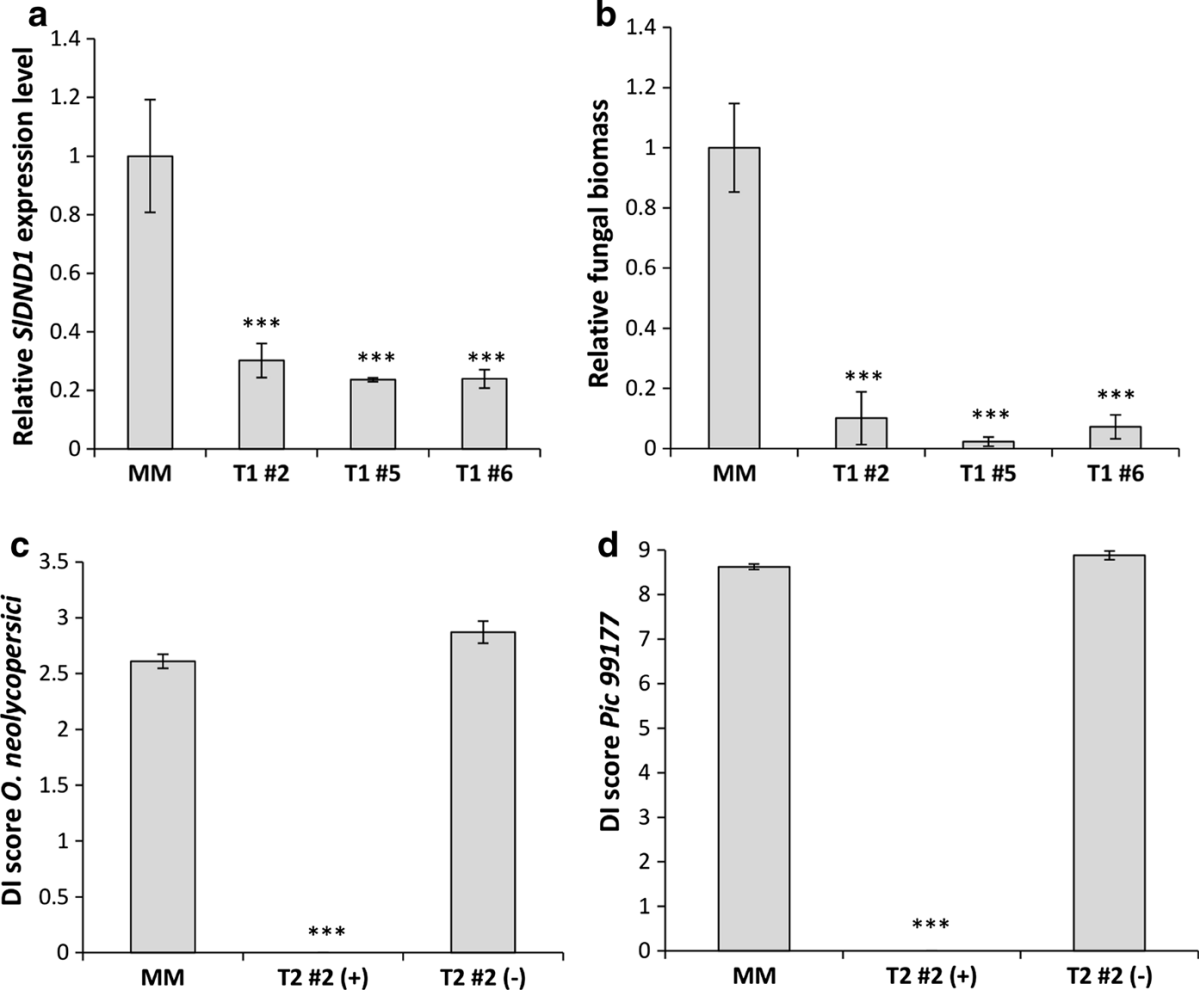
Powdery mildew and late blight resistance in tomato by impairment of *SlDND1*. **a** Relative *SlDND1* transcript levels in plants of untransformed cultivar Moneymaker (MM) and three independent T1 plants (T1 #2, #5 and #6) transformed with a silencing construct specifically targeting *SlDND1.* **b** Relative fungal biomass of *Oidium neolycopersici* on MM and the three T1 Plants in **a** measured 9 days post inoculation (dpi). **c** Disease index (DI) scores of T2 progeny (T2#2 family derived from T1#2) harbouring a *SlDND1* silencing construct (+, n = 13) or not (−, n = 1) at 10 dpi with *O. neolycopersici*. DI scores range from 0 (resistant) to 3 (susceptible). **d** DI scores of the same plants as in **c** but inoculated with *Phytophthora infestans* isolate Pic99177. DI scores range from 0 (resistant) to 9 (susceptible) and represent a mean of five leaflets of the 3rd leaf (from the *bottom*) per plant. After harvesting the 3rd leaf for testing with *P. infestans*, the same plant was then inoculated with *O. neolycopersici.* For both powdery mildew and late blight, a significant reduction in DI scores was observed on the T2 plants harbouring a silencing construct (+) compared to plants of MM and T2 plants not harbouring a silencing construct (−). *Asterisks* indicate degree of significance compared to MM (***p < 0.001)

Tomato plants of cultivar Moneymaker (MM) were transformed with RNAi::DND1A and the expression level of *SlDND1* was measured in leaves of 5-week-old T1 plants. Among ten transformants, three T1 plants (T1#2, #5 and #6) showed about 80 % reduction of *SlDND1* expression, compared to MM (Fig. 4a). These three independent *SlDND1* silenced T1 transformants and untransformed MM were multiplied by cuttings. Ten cuttings of each T1 plant were inoculated with *O. neolycopersici*. To assess the disease severity, fungal biomass was measured. Compared with MM, significant lower amounts of fungal biomass were detected in these three T1 plants (Fig. 4b).

These T1 plants were maintained for seed production. Unfortunately, due to low male fertility of the T1 transformants, selfed progeny could only be obtained from one T1 plant; T1#2. The resulting T2 family (T2#2) was inoculated with *O. neolycopersici*, and Disease Index (DI) was scored at 10 dpi. The T2#2 plants harbouring a silencing construct (+) showed significantly lower DI score compared to untransformed MM plants and T2#2 plants not harbouring a silencing construct (−) (Fig. 4c). The same T2#2 plants were tested by DLA with spores of *P. infestans* isolate Pic99177. A significant difference in DI scores at 6 dpi was observed between T2#2 plants harbouring (+) and not carrying (−) the silencing construct (Fig. 4d). Thus, silencing of *SlDND1* in tomato resulted in resistance to powdery mildew (*O. neolycopersici*) and *P. infestans* showing agreement with the results obtained in potato.

### Silencing of DND1 leads to side effects

**Fig. 5:**
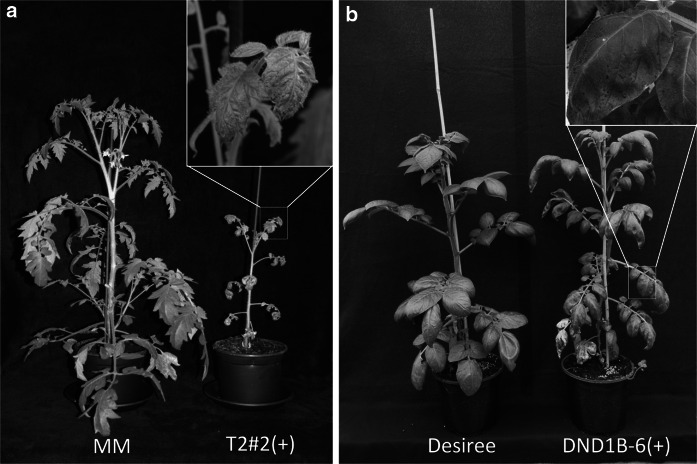
Morphological alterations observed on tomato and potato by impairment of *DND1* orthologs. **a** Picture of tomato cultivar Moneymaker (MM) compared to *SlDND1*-silenced MM transformant T2#2(+). Both plants were five weeks old. The T2#2(+) plant shows a dwarf phenotype and autonecrotic spots on all leaves. **b** Photo of a potato cultivar Desiree plant compared to *StDND1*-silenced Desiree transformant DND1B-6(+). The DND1B-6(+) plant shows similar growth vigour as Desiree. Autonecrotic spots are visible on older leaves only, not on younger leaves

Strong RNAi silencing of *SlDND1* in tomato, resulted in two phenotypic side effects, dwarfing and necrosis (Fig. 5a). Autonecrosis (spontaneous cell death without pathogen infection) was observed on non-inoculated leaves of tomato transformants. Autonecrosis was also found on leaves of *StDND1*-silenced potato plants (Fig. 5b) However, it was much less severe than that in the tomato *SlDND1*-silenced transformants. In tomato, necrotic spots were present on both old and young leaves, while in potato necrosis occurred only on older leaves at greenhouse conditions. When the potato *StDND1*-silenced transformants were grown in a screen cage under outdoor conditions the plants looked healthy. Only after 3 months, very small autonecrotic spots developed on the older leaves (Supplementary Fig. 8).

### Expression of defense-related gene StPR1 in DND1-silenced potato

In the *Arabidopsisdnd1* mutant an elevated level of salicylic acid (SA) and a constitutively high expression level of *PR1* (pathogenesis related protein 1) have been reported (Clough et al. 2000; Ahn 2007). To gain some insight in the mechanism of resistance in *DND1*-silenced potato transformants transcript levels of *StPR1* were monitored in Desiree and the *DND1* transformants by qRT-PCR. First, the expression level was measured in all eight potato transformants studied without pathogen infection. All six well-silenced transformants (+) showed a high constitutive expression level compared with Desiree, while the weak-silenced transformants (−) showed the same low level of expression as Desiree (Supplementary Fig. 9a). Next, the influence of inoculation with *P. infestans* on *StPR1* expression level was measured in three selected transformants; two well-silenced (+) and one weak-silenced (−) transformant compared with Desiree. Expression was measured in both mock-inoculated and *P. infestans*-inoculated leaves. As is shown in Supplementary Fig. 9b the expression of *StPR1* was highly up-regulated 6 days after *P. infestans* inoculaton in both the transformants and Desiree. However, the level of expression was significantly higher in *DND1* well-silenced (+) transformants compared with Desiree and weak-silenced (−) transformants. Thus, both the constitutive and the pathogen-induced level of expression of *StPR1* was increased after silencing *StDND1*.

## Discussion

### Loss of susceptibility factor as an alternative disease resistance type

To combat *P. infestans*, a highly flexible pathogenic oomycete, it is important to identify new types of resistance, in addition to the classical *R* gene-based resistance (Vleeshouwers et al. 2008). Recessive resistance, not associated with HR reaction, is widely used in crops to provide resistance against viruses, such as resistance to *PVY* in pepper or to *Bymoviruses* in barley (e.g. barley yellow mosaic virus and barley mild mosaic virus) (Ruffel et al. 2002; Stein et al. 2005). Recently, this kind of resistance was also reported against other pathogens, such as the recessive resistance alleles *rk1* and *rhg1* against root-knot nematodes in cowpea and cyst nematodes in soybean, respectively (Das et al. 2008; Afzal et al. 2009). The observation in *Arabidopsis* that loss-of-function mutations in many more *S* genes can lead to resistance to pathogens is remarkable. Evidence is accumulating that the susceptibility function of these *ArabidopsisS* genes is conserved across plant species. For example, down regulation of orthologs of *PMR4* and *MLO* leads to powdery mildew resistance in tomato (Bai et al. 2008; Huibers et al. 2013). More surprisingly, resistance resulting after silencing of orthologs of these *ArabidopsisS* genes in most cases appears to be effective against a wide range of pathogens. For example, the *Arabidopsisdmr1* gene was originally identified in a screen for resistance to downy mildew caused by *H. parasitica*, and silencing the tomato ortholog resulted in powdery mildew resistance (Huibers et al. 2013). The *Arabidopsis dnd1* mutant was previously reported to be resistant to bacterial, fungal and viral diseases (Yu et al. 1998). In this study, we show for the first time that the *DND1* gene can be considered as an *S* gene for late blight and powdery mildew diseases. It is likely that a susceptibility factor encoded by *S* genes is used by multiple pathogens to promote disease development. Therefore, the identified *ArabidopsisS* genes can be tested in crops to achieve broad-spectrum resistance via loss-of-function mutations and/or RNAi technique. Both approaches are possible in self-fertilizing diploid crops like tomato, while, in tetraploid potato, RNAi is the most realistic possibility. The advantage of the RNAi approach is that resistance obtained via RNAi silencing behaves as a dominant resistance trait, which is preferable for practical breeding programs especially of polyploid, vegetatively propagated crops. Very recently, it was shown that reduced expression of the putative *S* gene *SYNTAXIN*-*RELATED1* (*StSYR1*), an ortholog of *AtSYP121/AtPEN1* of *Arabidopsis*, decreased susceptibility to *P. infestans* in potato via the RNAi approach (Eschen-Lippold et al. 2012a, b).

### Fitness costs associated with loss of susceptibility factors can be plant-species dependent

*S* genes are expected to have important biological functions in plants besides being a susceptibility factor (Pavan et al. 2010). For example, the rice *Xa13* gene is required for both the growth of *X. oryzae* and for pollen development (Chu et al. 2006). Thus, resistance to pathogens achieved by silencing of *S* genes is potentially associated with negative side effects on other agronomic traits, as shown by the severe dwarfing and autonecrosis in *DND1*-silenced tomato plants (Fig. 5a). Therefore, more phenotypic studies are needed to determine whether loss-of-function of *DND1* can lead to plants which are acceptable for agriculture/horticulture. Such plants can then also be used as a new source of resistance in breeding. This resistance is inherited recessively after mutation induction or dominantly after RNAi transformation. Among the *StDND1*-silenced potato transformants we could already select plants with a good level of resistance combined with an almost normal phenotype in the screen cage (Supplementary Fig. 8a). These transformants were obtained with an RNAi construct containing the strong 35S promoter. We will try using a native promoter for the expression of the RNAi construct to investigate whether it is possible to obtain plants with a high level of late blight resistance but without fitness costs.

The phenotypic effects of dwarfing and autonecrosis were also described for the original *dnd1* mutant in *Arabidopsis* (Clough et al. 2000). In *DND1*-silenced tomato plants, it was shown that male fertility was decreased and thus offspring after selfing could not be obtained from all transformants. In *Arabidopsis*, DND1/CNGC2 has been reported to play a role in flowering timing and fertility (Chaiwongsar et al. 2009; Chin et al. 2013). Fortuna et al. (2015) reported that the role of DND1 in flowering timing is independent from SA accumulation, and independent from its role in pathogen defense. Further investigations are necessary to determine whether disease resistance can be achieved in tomato without reduced fertility by decreased expression level or altered functionality of *SlDND1*.

Interestingly, a much lower level of autonecrosis was found in *DND1*-silenced potato plants compared to tomato, although they were obtained using the same silencing constructs. Surprisingly, dwarfing was not observed at all in *DND1*-silenced potato plants under the tested growth conditions, indicating that the fitness costs associated with loss-of-function of *S* genes seems to depend on the plant species. One explanation for this apparent difference between tomato and potato could be the higher ploidy level combined with a high level of heterozygosity (multi-allelism) of potato that could provide a better genetic buffer against pleiotropic, multi-genic and multi-allelic effects at expression level than in diploid homozygous tomato (Gebhardt 2013). Alternatively, the fitness costs after knocking-out plant *S* genes may depend on the crop species, as was shown for the *mlo* mutation. In both *Arabidopsis* and barley, *mlo* mutants showed early senescence, which is however not observed in the natural *ol2* mutant of tomato (Bai et al. 2008). Since yield is the final measure of fitness costs, it will be necessary to test *StDND1* silenced potato plants in the field under natural environmental conditions.

### Mechanisms of dnd1-mediated resistance

The *DND1* gene was identified in *Arabidopsis* with a screen designed to discover additional components of the *avrRpt2*-*RPS2* disease resistance pathway (Yu et al. 1998, 2000). Compared to the wild type Col-0, the loss-of-function mutant *dnd1* displayed an inability to produce a HR in response to avirulent isolates of *Pseudomonas syringae* pv. *glycinea*. In addition, the *dnd1* mutant showed reduced growth, a constitutively elevated level of expression of the *PR1* gene, HR-like spontaneous lesions without pathogen infection, and resistance to unrelated pathogens including *H. parasitica, Botrytis cinerea* (Clough et al. 2000) and *Pectobacterium carotovorum* (the bacterial agent causing soft rot disease on Korean cabbage, Ahn 2007). It may be argued that *dnd1*-associated autoimmune responses (such as autonecrosis and dwarfing) result in stressed plants, which in turn may enhance resistance to pathogens. However, we have shown that the *DND1*-silenced potato transformants show resistance to late blight and powdery mildew, a constitutively elevated level of expression of the *StPR1* gene and small autonecrotic lesions, but no reduced growth. Furthermore, the severity of autonecrosis in potato was dependent on environmental conditions. This has also been reported for the *dnd1* mutant in *Arabidopsis* (Clough et al. 2000; Moeder et al. 2011). However, we observed that the level of pathogen resistance was not influenced by environmental conditions, at least in the tuber slice assay. In addition, there is evidence showing that defence-related signalling pathways are required for resistance in the *dnd1* mutants, but not for autoimmune phenotypes (Clough et al. 2000; Jirage et al. 2001; Jurkowski et al. 2004). Thus, the resistance resulting from loss-of-function of *DND1* is not necessarily associated with severe autoimmune phenotypes.

The *DND1* gene encodes a cyclic nucleotide-gated cation channel (CNGC) belonging to a large family consisting of 20 members in *Arabidopsis* (Chin et al. 2013). The CNGCs have a role in conducting Ca^2+^ into plant cells and are involved in various physiological processes (Sherman and Fromm 2009). The precise role of *AtCNGC2* (*DND1*) in plant defence responses remains elusive, although evidence has been obtained that activation of multiple defence pathways are required for the broad-spectrum resistance in *dnd1* mutants (Genger et al. 2008; Moeder et al. 2011). For example, the resistance to *P. syringae* and *H. parasitica* in *dnd1* mutants is dependent on SA accumulation (Genger et al. 2008). Further, the PR1 protein has been shown to have inhibitory activity against *P. infestans* (Niderman et al. 1995). Thus, the highly elevated *PR1* expression in DND1-silenced plants may lead to increased PR1 protein content, which can play an important role in resistance against *P. infestans*. Resistance to *B. cinerea* could be abolished by disrupting ethylene signalling (Genger et al. 2008). Further experiments are needed to study the resistance mechanisms associated with the resistance to late blight and powdery mildew in *DND1*-silenced potato and tomato plants.

### Perspectives of S genes for resistance breeding

Deployment of plant *S* genes in breeding crops with resistance to pathogens and pests is a relatively new approach as proposed by us in 2010 (Pavan et al. 2010). In the past few years, more and more examples have arisen, where resistance can be achieved by altering plant genes that are required by pathogens to establish a compatible interaction with hosts (reviewed in Van Schie and Takken 2014). Here, we show that *dnd1*, which plays a pivotal role in disease resistance, may be an interesting gene in resistance breeding of crops. Although we observed negative side effects upon gene silencing via RNAi, especially in tomato, it may be possible to minimise these fitness costs by investigating sufficient numbers of RNAi transformation events, by transformation of different genotypes and/or by sexual crossing followed by selection for offspring plants with normal phenotypes in the presence of pathogen resistance. The high level of heterozygosity and multi-allelism in potato could open up the possibility to select for a more favourable genetic background when using this approach. As shown in this study, silencing *DND1* resulted in less fitness costs in potato than in tomato (Fig. 5). In diploid crops like tomato, variation in loss-of-function mutations can be obtained by assessing natural mutations and mutations induced by chemical mutagenesis, such as EMS (Emmanuel and Levy 2002; Minoia et al. 2010). In potato, the approach using mutation induction is possible, as earlier shown for the recessive *amf* mutant with amylose-free starch (Hovenkamp-Hermelink et al. 1987). However, breeding at the tetraploid level with recessive traits is complicated, although it has recently become more realistic by using allele-specific markers. This approach would be much more appropriate after realisation of the method of hybrid seed propagated potato varieties which are based on a cross between self-compatible diploid homozygous parental lines (Lindhout et al. 2011). Nowadays, new techniques are available to enable the discovery and design of superior allele variants, which combine favourable disease resistance levels with no or less detrimental side effects. Such advanced technologies include TILLING (targeting induced local lesions in genomes); site-directed mutagenesis using Zinc-finger nucleases (Colbert et al. 2001), TALEN (Transcription activator-like effector nuclease)-based gene editing for allele design (Lloyd et al. 2005; Li et al. 2012) and CRISPR-/Cas9 (clustered regularly interspaced short palindromic repeats–associated nuclease Cas9) (Belhaj et al. 2013; Xie and Yang. 2013). For example, the TALEN technology was successfully used to edit the rice gene *Os11N3* for bacterial blight susceptibility resulting in a specific allele altering this *S* gene function (Li et al. 2012). Combining TALEN and CRISPR-/Cas9 techniques, Wang et al. (2014) simultaneously edited three homeoalleles of the *MLO* gene in hexaploid wheat providing resistance to powdery mildew. Taken together, these approaches hold substantial promise in defining and deploying altered host *S* genes for durable resistance in controlling plant diseases (Dangl et al. 2013).

## Electronic supplementary material

Supplementary material 1 (DOC 3620 kb)

Supplementary material 2 (MP4 145970 kb)
