# Supplementary material for: Down-regulation of ArabidopsisDND1 orthologs in potato and tomato leads to broad-spectrum resistance to late blight and powdery mildew
Source: Transgenic Res. 2015 Nov 17;25:123–38. doi: 10.1007/s11248-015-9921-5 (PMC4762934; doi:10.1007/s11248-015-9921-5)
Supplement: Supplementary file 1 — Supplementary material 1 (DOC 3620 kb) [file 11248_2015_9921_MOESM1_ESM.doc]

**Electronic Supplementary Material**

**Transgenic Research**

**Down-regulation of *Arabidopsis* *DND1* orthologs in potato and tomato leads to broad-spectrum resistance to late blight and powdery mildew**

Kaile Sun^1^, Anne-Marie A. Wolters^1^, Annelies E.H.M. Loonen^1^, Robin P. Huibers^1^, René van der Vlugt^2^, Aska Goverse^3^, Evert Jacobsen^1^, Richard G.F. Visser^1^, Yuling Bai^1, *^

^1^ Wageningen UR Plant Breeding, Wageningen University and Research Centre, Droevendaalsesteeg 1, 6708 PB Wageningen, The Netherlands

^2^ Laboratory of Virology, Wageningen University and Research Centre, Droevendaalsesteeg 1, 6708 PB Wageningen, The Netherlands

^3^ Laboratory of Nematology, Wageningen University and Research Centre, Droevendaalsesteeg 1, 6708 PB Wageningen, The Netherlands

^*^ Corresponding author

e-mail: bai.yuling@wur.nl

tel: +31 317 482136

fax: +31 317 483457

| **Supplementary Table 1** Primers used in this study | | | | |
| --- | --- | --- | --- | --- |
| Primer name | Sequence (5' to 3') | Gene/Accession | Used for |  |
| Fw-DND1A | caccTGTCTTCTCACCAAGACGTCCGC | Solyc02g088560 | RNAi:: DND1A cloning | |
| Rv-DND1A | CGATGTGTGCCGGCTGCGAC |  |  | |
| Fw-DND1B | caccTGATGAGGACTAGCCGTCCCAC | Solyc02g088560 | RNAi:: DND1B cloning | |
| Rv-DND1B | AGCAAATAATTCCCGGGCCCTTC |  |  | |
| Fw-SlDND1-qPCR | GGGCCGTTCGGGCGTGTATT | Solyc02g088560 | Determining relative transcript levels | |
| Rv-SlDND1-qPCR | CCACCGCGGCCGATGGATAA |  |  | |
| Fw-StDND1-qPCR | GTTCGGGCGTGTATTAGACC | Sotub02g034320 | Determining relative transcript levels | |
| Rv-StDND1-qPCR | GAATCACCGTGACGATAGCC |  |  | |
| Fw-SlEF1a | ATTGGAAACGGATATGCCCCT | Solyc06g005060 | Normalisation in qRT-PCR assays | |
| Rv-SlEF1a | TCCTTACCTGAACGCCTGTCA |  |  | |
| Fw-StEF1a | ATTGGAAATGGATATGCTCCA | Sotub06g010680 | Normalisation in qRT-PCR assays | |
| Rv-StEF1a | TCCTTACCTGAACGCCTGTCA |  |  | |
| Fw-On | CGCCAAAGACCTAACCAAAA | EU047564 | Quantification of *Oidium neolycopersici* | |
| Rv-On | AGCCAAGAGATCCGTTGTTG |  |  | |
| Fw-StPR1a | TGGTGATTTCACGGGGAGGG | AJ250136 |  | |
| Rv-StPR1a | CGAACTGAGTTGCGCCAGAC |  |  |  |

| **Supplementary Table 2** Background information of *Phytophthora infestans* isolates used in this study | | | | | | | |
| --- | --- | --- | --- | --- | --- | --- | --- |
| Isolate | Race | Mating type | Origin | | | Provider of isolate | Reference |
|  |  |  | Year of collection | Country | Source |  |  |
| Pic99177 | 1.2.3.4.7.9.11 | A2 | 1999 | Mexico | *So**lanum stoloniferum* | Kessel, WUR, Netherlands | Flier et al.2002 |
| Pic99189 | 1.2.5.7.10.11 | unknown | 1999 | Mexico | *Solanum stoloniferum* | Kessel, WUR, Netherlands | Flier et al.2002 |
| EC#1 | 1.3.4.7.10.11 | unknown | unknown | Ecuador | unknown | Birch, SCRI, Scotland | Armstrong et al.2005 |
| USA618 | 1.2.3.6.7.10.11 | A2 | unknown | Mexico | Potato crop | Fry, Cornell, USA | Goodwin et al. 1994 |

**References**

Armstrong MR, Whisson SC, Pritchard L, Bos JIB, Venter E, Avrova AO, Rehmany AP, Böhme U, Brooks K, Cherevach I, Hamlin N, White B, Fraser A, Lord A, Quail MA, Churcher C, Hall N, Berriman M, Huang S, Kamoun S, Beynon JL, Birch PRJ (2005) An ancestral oomycete locus contains late blight avirulence gene *Avr3a*, encoding a protein that is recognized in the host cytoplasm. Proc Natl Acad Sci USA  102:7766–7771

Flier WG, Grünwald NJ, Kroon L, van den Bosch TBM, Garay-Serrano E, Lozoya- Saldana H, Bonants PJM, Turkensteen LJ (2002) *Phytophthora ipomoeae* sp. Nov., a new homothallic species causing leaf blight on *Ipomoea longipedunculata* in the Toluca Valley of central Mexico. Mycol Res 106:848-856

Goodwin S, Cohen BA, Fry WE (1994) Planglobal distribution of a single clonal lineage of the Irish potato famine fungus. Proc Natl Acad Sci USA 91:11591- 11595

**Supplementary Fig. 1** Identification of *DND1* orthologs in tomato and potato. **a** Sequence alignment of *Arabidopsis* AtDND1, tomato SlDND1 and potato StDND1 protein sequences. The numbers on the left indicate the amino acid position. Identical residues in all these proteins are shown in a dark blue background. Dots indicate gaps introduced for optimal alignment. The *SlDND1* (*Solyc02g088560)* and *StDND1* (*Sotub02g034320)* genes are regarded as *DND1* orthologs in tomato and potato, respectively. **b** Phylogenetic tree of *Arabidopsis* DND1 and other *Arabidopsis* AtCNGC members, together with tomato SlDND1 (*Solyc02g088560)* and potato StDND1 (*Sotub02g034320)*.


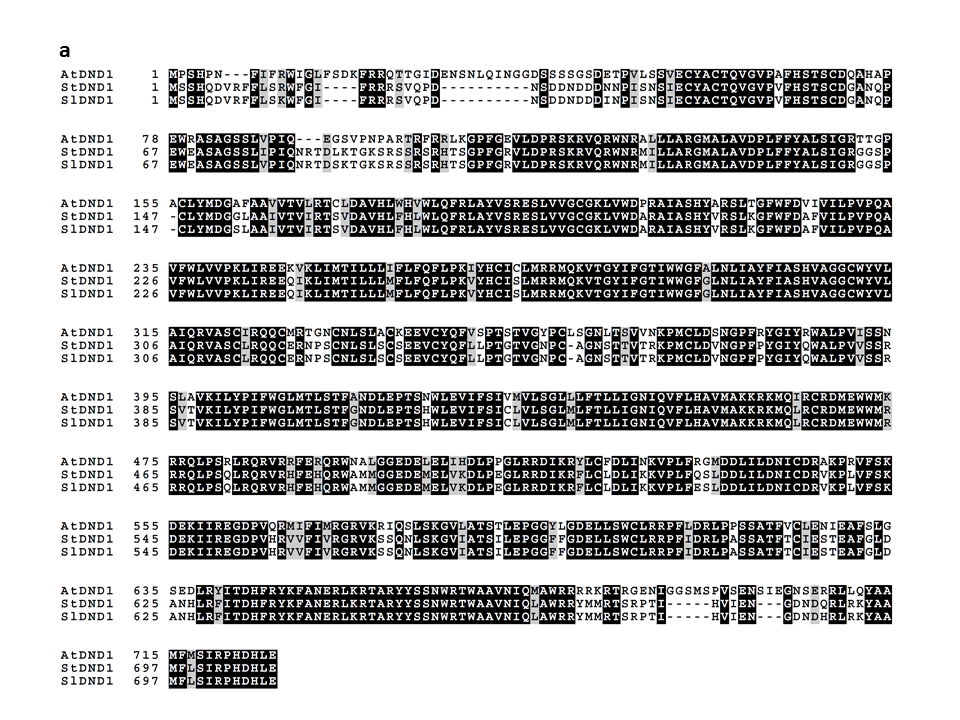


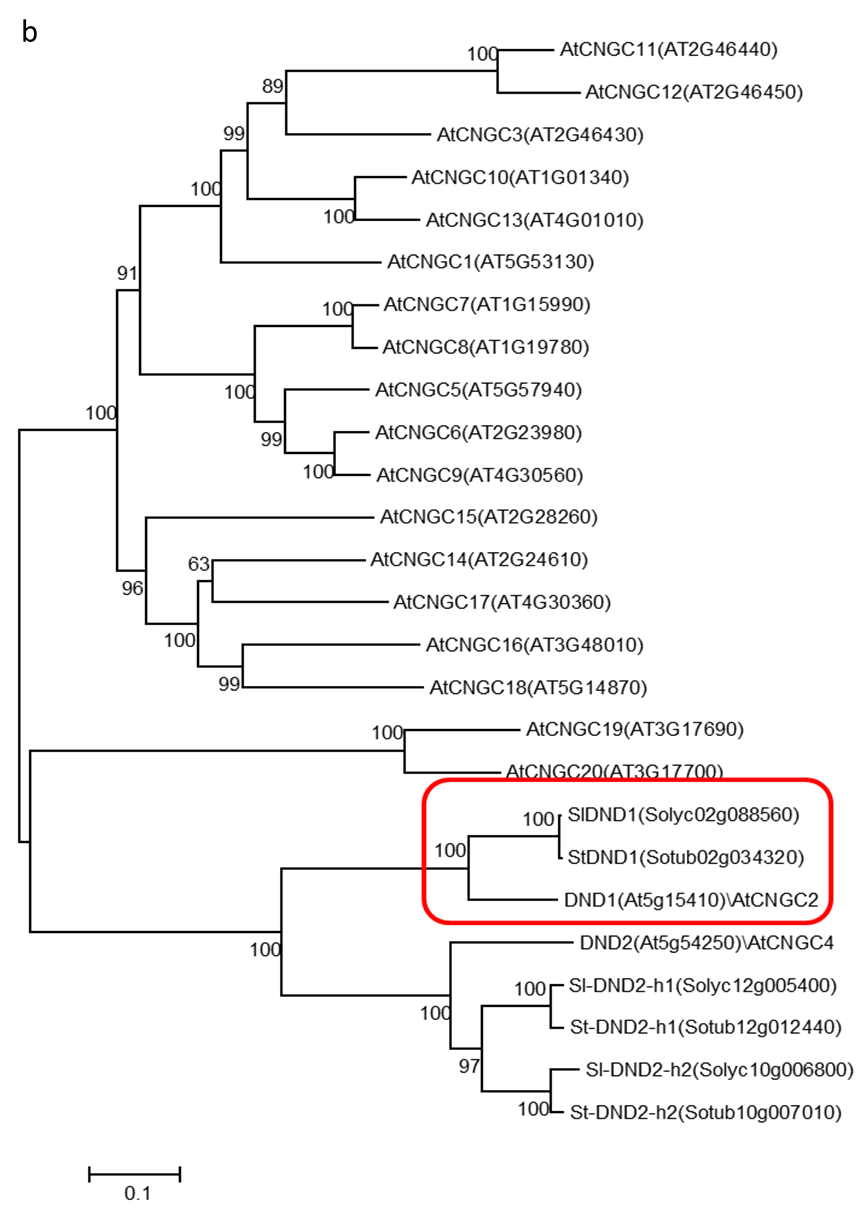


**Supplementary Fig. 2.** Transcript profiles of *DND1* in major tissues of tomato (**a**, *SlDND1*) and potato (**b**, *StDND1*). The relative expression level of *DND1* in leaf, root, flower, fruit (tomato only), and tuber (potato only) samples were evaluated by qPCR. For each sample, the transcript level of tomato or potato gene *EF1a* was used as reference. For both tomato and potato, the relative expression level in leaves was defined as 1. Data indicate the mean of 3 biological replicates with error bars representing the standard error. Asterisks indicate degree of significance compared to MM or Desiree leaves, respectively. (*p<0.05, ***p<0.001). **c** and **d** RNAseq data of *SlDND1* (*Solyc02g088560*) and *StDND1* (*Sotub02g034320*), respectively.


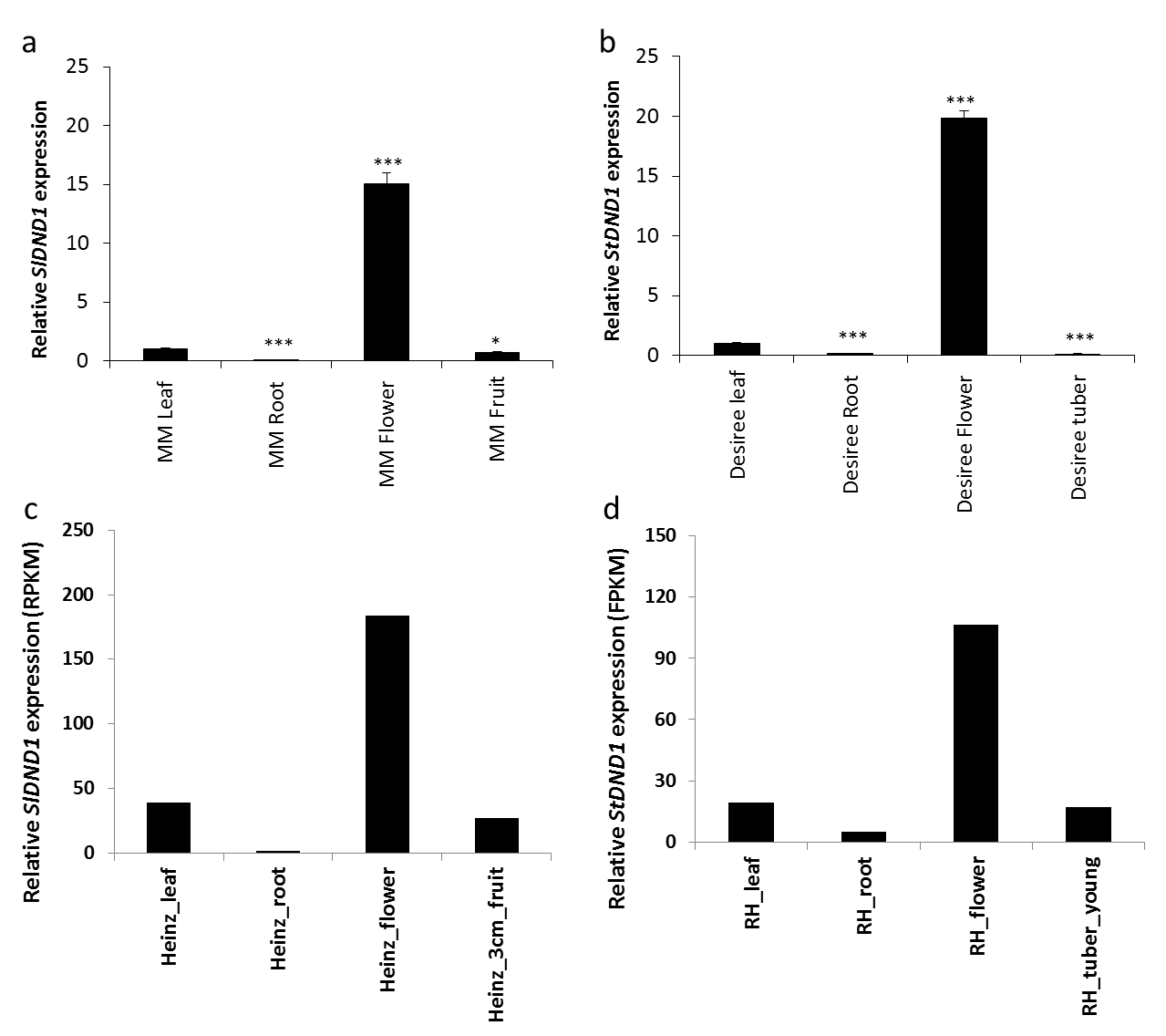


**Supplementary Fig. 3** Information of RNAi silencing constructs and silencing effects on RNAi potato transformants. **a** Location of the targeting regions of the two RNAi constructs in *SlDND1* (*Solyc02g088560*) and *StDND1* (*Sotub02g034320*). **b** Alignment of RNAi fragments for the *DND1A* and *DND1B* genes in tomato and potato. **c** Relative expression level of *StDND1* in leaves of Desiree and 16 independent potato RNAi::*DND1* transformants (DND1A-1, 3, 5, 6, 8, 11, 16 , 17 and DND1B-1, 3, 4, 6, 8, 11, 12, 13). Plants marked + (well-silenced) or – (weak-silenced) were used in the following experiments.


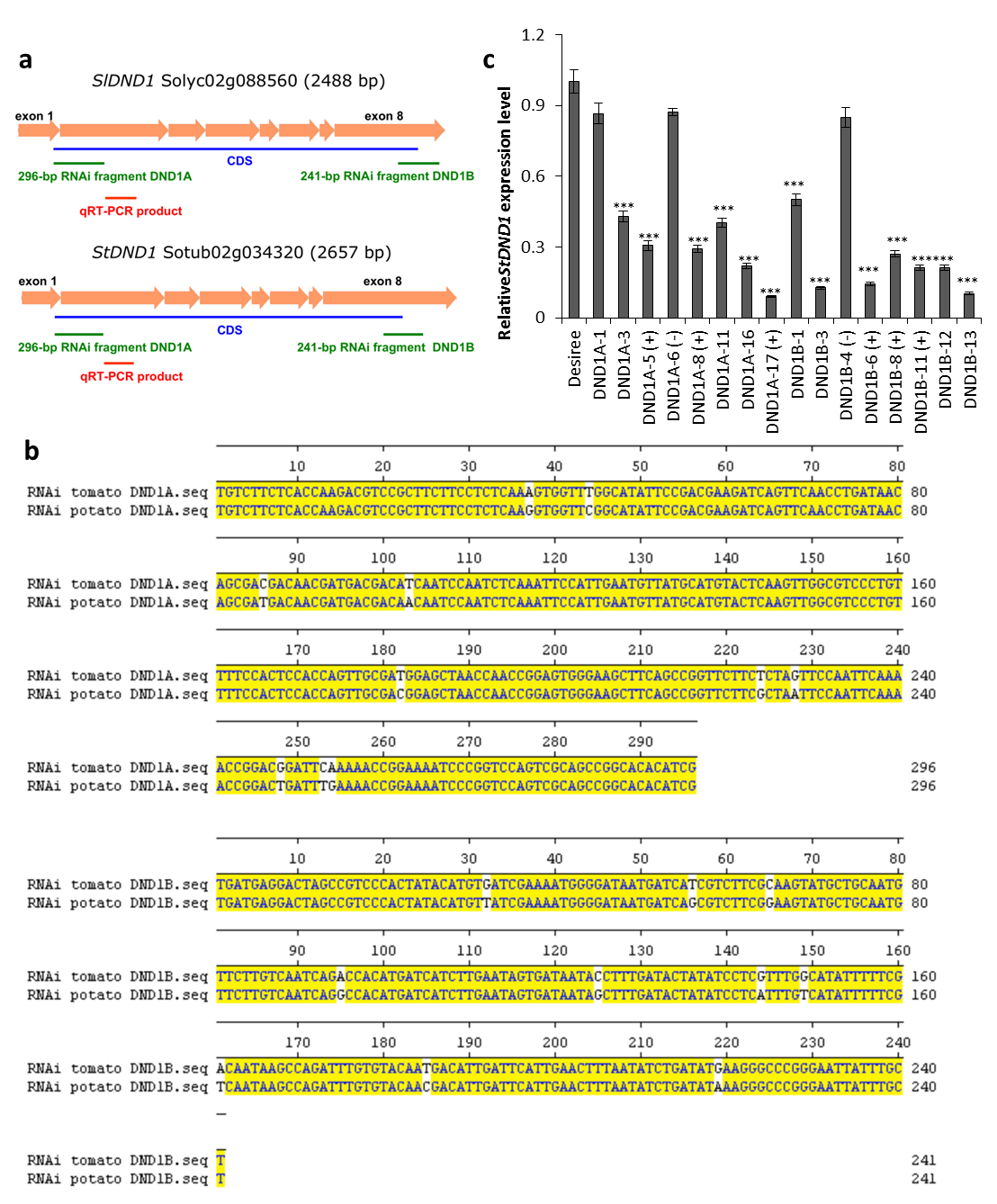


**Supplementary Fig. 4** Late blight resistance in potato tubers from the screen cage by silencing *StDND1*. Tubers were harvested from Desiree, A13-013 and eight independent potato RNAi::*DND1* plants (DND1A-5, 6, 8 and 17, DND1B-4, 6, 8 and 11). Numbers of sporangia on tubers infected with *Phytophthora infestans* isolate Pic99189. For each transformant, four plants were tested (one tuber per plant). Asterisks indicate degree of significance compared to Desiree plants (***p<0.001).


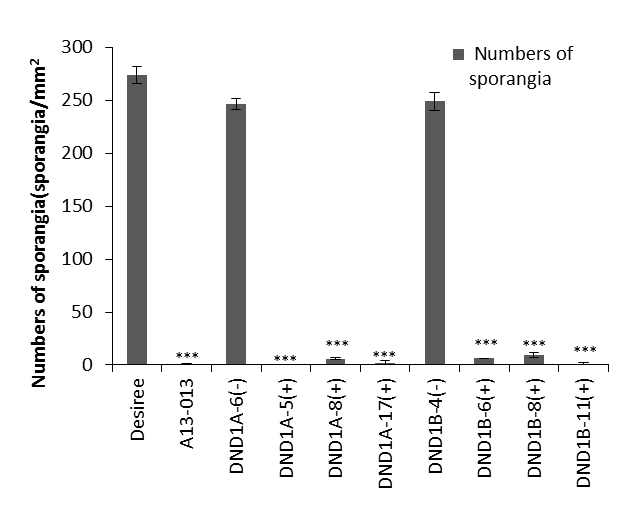


**Supplementary Fig. 5** Powdery mildew resistance in potato by impairment of *StDND1*. **a** Photo of a leaf from a Desiree (left) plant compared to a leaf from a *StDND1-*silenced plant (DND1B-11, right). Plants were grown in the greenhouse and naturally infected with powdery mildew. **b** PCR fragment of 621 bp amplified with fungal ITS primers ITS5A and ITS4 using DNA isolated from the powdery mildew-infected leaves. **c** Disease index (DI) scores of the same plants used in Fig. 1 after 6 weeks growth in the greenhouse. DI scores range from 0 (resistant) to 3 (susceptible). All three *StDND1* silenced (+) plants showed no fungal sporulation (DI=0) compared to the controls Desiree, A13-013 and *StDND1*-silenced (–) plants, which had a DI of about 2.5. **d** Fungal biomass quantification of powdery mildew on the same plants used in Fig. 1 after 8 weeks growth in the greenhouse. Relative fungal biomass was quantified by qRT-PCR with primers ITS5A and ITS4 using *StEF1a* as internal reference. All *StDND1* silenced (+) plants showed significant lower fungal biomass compared to the controls (Desiree and A13-013) and *StDND1*-silenced (–) plants. Data indicate the mean of 4 biological replicates with error bars representing the standard error. Asterisks indicate degree of significance compared to Desiree plants (***p<0.001).


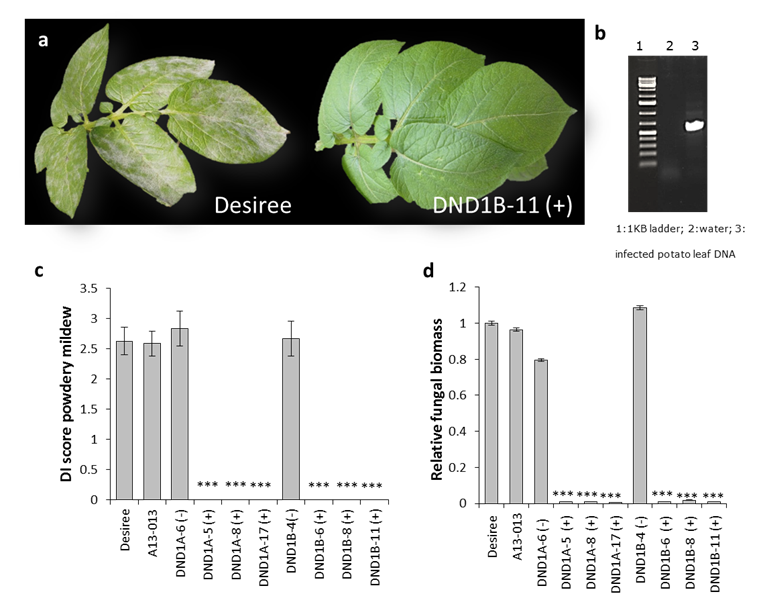


**Supplementary Fig 6** Quantification of PVY anti-serum in Desiree and *StDND1-*silenced plants. Data indicate the mean of four biological replicates with error bars representing the standard error.


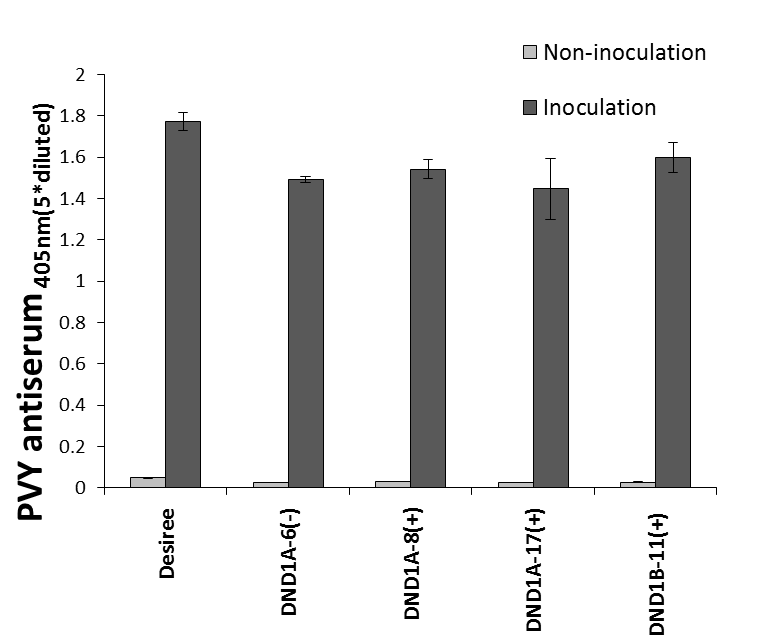


**Supplementary Fig 7** Number of nematode cysts formed on plant roots of Desiree, *StDND1-*silenced transformants and the resistant control genotype SH. The cyst nematode *Globodera rostochiensis,* line 19 was used. Data indicate the mean of five biological replicates with error bars representing the standard error.


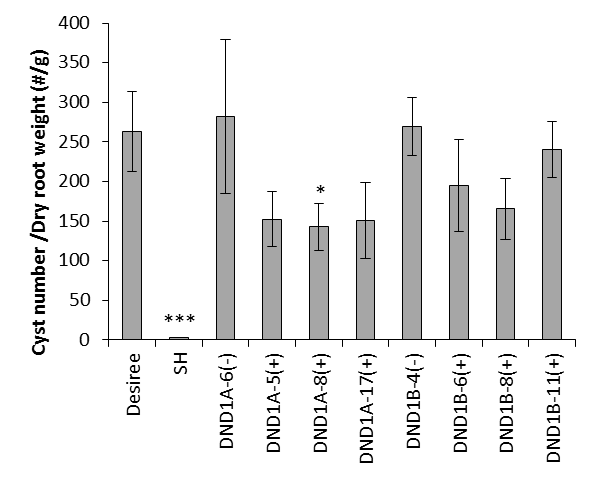


**Supplementary Fig 8** Phenotypes of the wild type Desiree and RNAi::StDND1 potato plants. **a** Plants grown in a screen cage. Pictures were taken two months after transfer to the screen cage. **b** Plants grown in the greenhouse. Picture was taken five weeks after transfer to the greenhouse. In the screen cage autonecrosis was less severe than in the greenhouse.


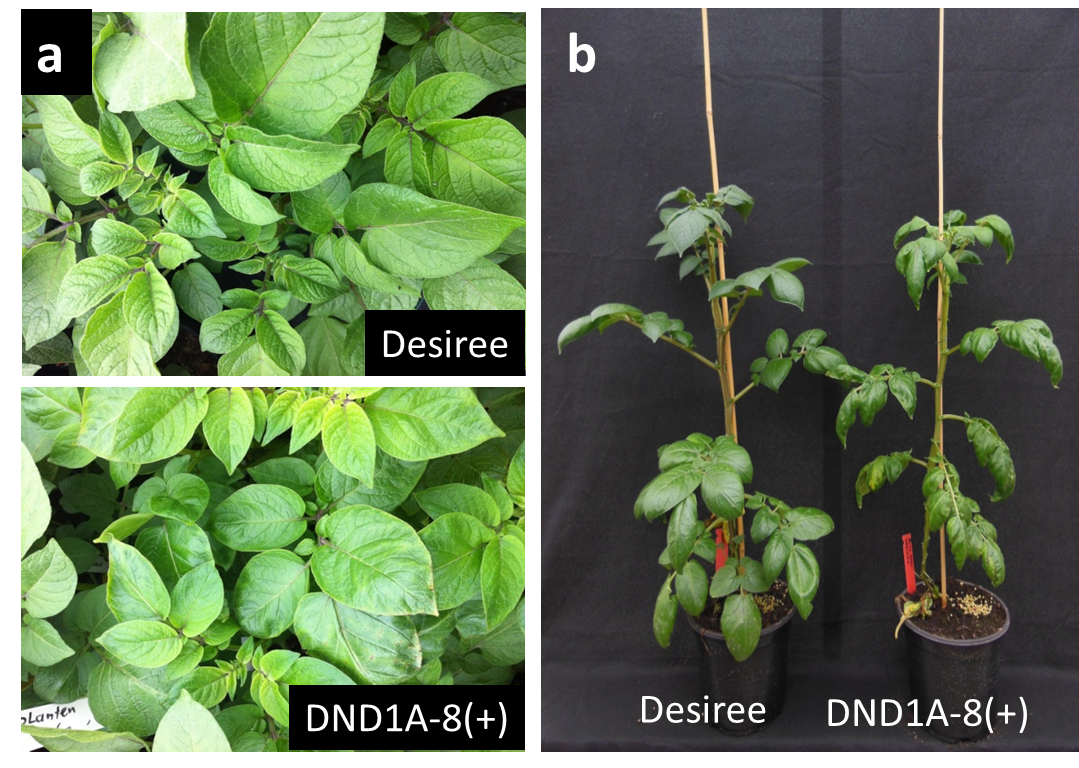


**Supplementary Fig 9** Relative *StPR1* (pathogenesis related protein 1) gene expression in Desiree and *StDND1-*silenced plants. **a** Expression level under non-inoculated conditions. **b** Comparison of expression level between mock and *P. infestans* isolate Pic99189-inoculated plants. For each assay, the expression level in non-inoculated or mock-inoculated Desiree was defined as 1. Data indicate the mean of 4 biological replicates with error bars representing the standard error. Asterisks indicate degree of significance compared to Desiree samples, non-inoculated in (A), or inoculated in (B) (***p<0.001).


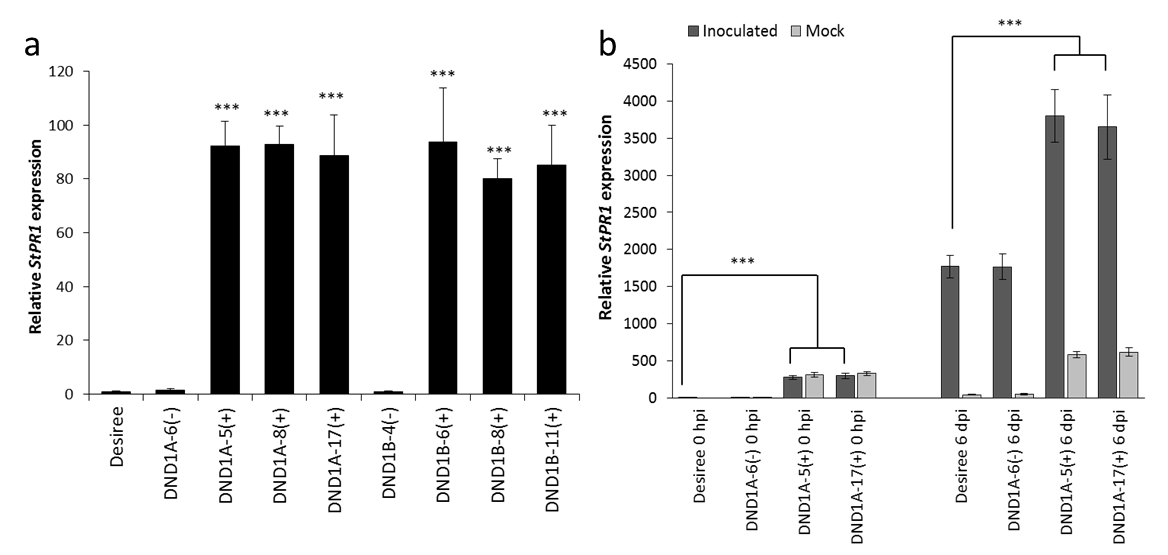


**Supplementary video 1** Time lapse video showing the development of *Phytophthora infestans* isolate Pic99189 on inoculated (I) and mock-inoculated (M) halves of detached leaves of wild type potato Desiree and transformants DND1A-6(-), DND1A-5(+) and DND1A-17(+). Times after inoculation are indicated.

25-06-2015 06:02, clear lesions can be observed on all Desiree and DND1A-6(-) I halves, but not on DND1A-5(+) and DND1A-17(+) I halves.

26-06-2015 10:25, late blight mycelium showing on the Desiree and DND1A-6(-) I halves, DND1A-5(+) and DND1A-17(+) leaflets are still clean and no infection.

27-06-2015 12:25, late blight mycelium covers the I halves of Desiree and DND1A-6(-) plants, no lesions and no mycelium growth on DND1A-5(+) and DND1A-17(+) leaflets.
